# Cell-based influenza vaccines: An effective vaccine option for under 60-year-olds

**DOI:** 10.3205/dgkh000476

**Published:** 2024-04-30

**Authors:** Barbara C. Gärtner, Dietmar Beier, Gunther Gosch, Klaus Wahle, Luise Wendt, Laura-Christin Förster, Kim J. Schmidt, Tino F. Schwarz

**Affiliations:** 1 Institute for Medical Microbiology and Hygiene – Saarland University Hospital, Homburg/Saar, Germany; 2 Saxon Committee on Vaccinations (SIKO), Chemnitz, Germany; 3 Kinderarztpraxis am Domplatz, Magdeburg, Germany; 4 Medical Faculty – University of Münster, Münster, Germany; 5 ias-Gruppe, Leipzig, Germany; 6 CSL Seqirus GmbH, Munich, Germany; 7 Xcenda GmbH, Hanover, Germany; 8 Institute of Laboratory Medicine and Vaccination Center – Klinikum Würzburg Mitte, Standort Juliusspital, Würzburg, Germany

**Keywords:** influenza, egg adaptation, enhanced influenza vaccine, real-world data, vaccination recommendation

## Abstract

**Aim:**

Seasonal influenza poses a significant burden of disease, affecting not only older adults but also individuals under the age of 60. It carries a high economic burden, mainly driven by influenza-associated productivity losses in the working population. Conventional egg-based influenza vaccines may have reduced effectiveness due to antigen adaptation in eggs. In contrast, cell-based influenza vaccines are less likely to be affected by such antigen adaptation. This review aims to present real-world data (RWD) comparing the effectiveness of quadrivalent cell-based (QIVc) and egg-based (QIVe) influenza vaccines over three consecutive seasons.

**Methods:**

A comprehensive review was conducted, analyzing RWD from retrospective cohort and case-control studies on the relative vaccine effectiveness (rVE) of QIVc versus QIVe during the 2017/18–2019/20 seasons.

**Results:**

This study included six retrospective cohort studies and one case-control study, with a combined total of approximately 29 million participants. A cohort study involving people aged ≥4 years during the 2017/18 season showed a statistically significant rVE of QIVc compared to QIVe in preventing influenza-like illness, with a value of 36.2%. QIVc demonstrated statistically significant superiority over QIVe in preventing outpatient and inpatient medical encounters as observed in two cohort studies conducted during the 2018/19 and 2019/20 seasons. The rVE of QIVc compared to QIVe was found to be 7.6% in individuals aged ≥4 years and 9.5% in individuals aged ≥18 years. Three additional cohort studies conducted between 2017/18–2019/20 reported a statistically significant improvement in rVE (5.3–14.4%) of QIVc compared to QIVe in preventing influenza-related hospitalizations and emergency department visits due to influenza in individuals aged 4–64 years. In a case-control study across all three seasons, QIVc showed statistically significantly higher effectiveness compared to QIVe in preventing test-confirmed influenza, with rVEs of 10.0–14.8%.

**Conclusions:**

RWD from the 2017/18–2019/20 seasons demonstrated that QIVc is more effective than QIVe in preventing influenza-related outcomes in individuals aged 4–64 years. Preferential use of cell-based influenza vaccines, as opposed to conventional egg-based vaccines, could reduce the burden of influenza-related symptoms on individuals and alleviate the economic impact on the German population under 60 years of age.

## Zusammenfassung

**Zielsetzung:**

Die saisonale Influenza verursacht nicht nur bei älteren Erwachsenen, sondern auch in der Bevölkerung <60 Jahre eine signifikante Krankheitslast. Diese geht mit einer hohen wirtschaftlichen Belastung einher, insbesondere bedingt durch Influenza-assoziierte Produktivitätsverluste der arbeitenden Gesellschaft. Konventionelle eibasierte Influenzaimpfstoffe können aufgrund von Ei-Adaptation eine beeinträchtigte Impfeffektivität aufweisen, während zellkulturbasierte Influenzaimpfstoffe seltener von Antigenanpassungen betroffen sind. Ziel dieses Reviews war die Darstellung von „Real-World“-Daten (RWD) zur Effektivität von quadrivalenten zellkulturbasierten (QIVc) vs. eibasierten (QIVe) Influenzaimpfstoffen über drei konsekutive Saisons.

**Methoden:**

Es wurde ein narrativer Review von RWD aus retrospektiven Kohorten- und Fall-Kontroll-Studien zur relativen Impfeffektivität (rVE) von QIVc vs. QIVe in den Saisons 2017/18–2019/20 durchgeführt.

**Ergebnisse:**

Es wurden sechs retrospektive Kohortenstudien und eine Fall-Kontroll-Studie eingeschlossen, die rund 29 Mio. Teilnehmer umfassten. Eine Kohortenstudie der Saison 2017/18 bei Personen ≥4 Jahre ergab eine statistisch signifikante rVE von QIVc vs. QIVe von 36,2% bei der Prävention Influenza-ähnlicher Erkrankung. QIVc war statistisch signifikant effektiver vs. QIVe bei der Prävention ambulanter und stationärer medizinischer Behandlungen in zwei Kohortenstudien der Saisons 2018/19 bzw. 2019/20 mit rVEs von 7,6% bei ≥4-Jährigen bzw. 9,5% bei ≥18-Jährigen. Drei weitere Kohortenstudien der Saisons 2017/18–2019/20 ergaben eine statistisch signifikant höhere rVE von QIVc vs. QIVe bei der Prävention Influenza-bedingter Hospitalisierungen/Besuchen der Notaufnahme bei 4–64-Jährigen (5,3–14,4%). Auch bei der Prävention testbestätigter Influenza war QIVc in einer Fall-Kontroll-Studie über alle drei Saisons statistisch signifikant effektiver mit rVEs von 10,0–14,8%.

**Schlussfolgerungen:**

RWD der Saisons 2017/18–2019/20 zeigten eine bessere Impfeffektivität von QIVc vs. QIVe bei der Prävention Influenza-bedingter Endpunkte bei Personen im Alter von 4–64 Jahren. Der präferenzielle Einsatz von zellkulturbasierten vs. konventionellen Influenzaimpfstoffen könnte zu einer Verringerung der Influenza-bedingten Krankheitslast und wirtschaftlichen Belastung in der deutschen Bevölkerung <60 Jahre führen.

## Introduction

In the 2022/23 season, influenza emerged as one of the most prevalent laboratory-confirmed respiratory diseases in Germany. This followed a relatively low number of laboratory-confirmed influenza cases in the 2020/21 season, primarily due to the measures implemented to control the COVID-19 pandemic [1], [2]. The 2022/23 season was characterized by an early onset and early peak in calendar week (CW) 50/2022 [1]. A total of 292,625 laboratory-confirmed influenza cases, 42,562 hospitalizations, and 1,028 deaths associated with influenza infection were reported to the Robert Koch Institute (RKI) from CW 40/2022 to 21/2023. This magnitude of the influenza outbreak was comparable to severe seasons before the COVID-19 pandemic.

The relatively low number of deaths attributed to laboratory-confirmed influenza within the context of the COVID-19 pandemic does not accurately reflect the true impact of influenza morbidity and mortality. A more comprehensive assessment of mortality is provided by the excess mortality figures calculated by the RKI, with estimates reaching as high as 25,100 deaths during the severe 2017/18 season (conservative estimate), representing the highest number in the past 30 years [3]. 

Annually, around 5–20% of the population becomes infected during the seasonal influenza wave [4]. While individuals aged ≥60 years are particularly vulnerable to influenza-related hospitalizations and deaths due to chronic illness and immune senescence [2], a significant proportion of infections and associated physician visits are also recorded in people aged <60 years [2], [4]. Notably, children experience a high morbidity rate and substantial mortality [5]. 

### Burden of disease in adults <60 years

During the 2016/17–2018/19 seasons, individuals aged 35 to 59 years represented a large proportion (30–38%) of laboratory-confirmed influenza cases reported to the RKI [2]. Adults <60 years significantly contribute to the economic burden through work incapacity related to influenza [6]: Work absences of adult patients with clinically diagnosed influenza account for the majority of total societal costs in Germany [7]. In addition, the high burden of illness in children can lead to lost working hours for caring parents [5] and significantly increase absenteeism and the associated costs of lost productivity.

### Burden of disease in children and adolescents

Children and adolescents have a high infection rate and, unlike COVID-19, are the primary carriers of the virus to adults and the elderly [4], [8]. In particular, young children are at an increased risk of severe illness. Alongside the elderly population, they also experience a higher frequency of influenza-related hospitalizations. These hospitalizations are primarily caused by secondary complications (e.g., secondary bacterial pneumonia, acute otitis media, and sinusitis) [4], [8], which can lead to fatal outcomes. However, there is limited data on influenza mortality rates among children in Germany. In contrast to adults <60 years of age, the majority of the total societal costs associated with influenza in children and adolescents stem from direct costs related to physician visits and hospitalizations [7]. Costs resulting from work absenteeism of parents caring for sick children are not considered.

### Influenza prevention in the population <60 years of age

The Standing Committee on Vaccination (STIKO) in Germany recommends an annual standard vaccination against influenza for all persons aged ≥60 years [9]. For individuals aged ≥6 months to <60 years, vaccination is recommended for pregnant women, those with an increased health risk due to an underlying disease, residents of retirement and nursing homes, caregivers of people at risk, and people with an occupational risk (e.g., medical staff, facilities with extensive public traffic).

Unlike the STIKO, the Saxon Committee on Vaccinations (SIKO) has been recommending the annual standard vaccination against influenza for children from the age of 6 months, adolescents, and adults since 2010 [10]. The SIKO recommendation specifically applies to the federal state of Saxony. Additionally, the highest health authorities in all federal states recommend influenza vaccinations beyond the STIKO recommendation, irrespective of age [11]. 

### Vaccination rates in individuals <60 years with vaccination recommendation

Based on billing data from the Associations of Statutory Health Insurance Physicians (KV), which covers 85% of the population, and surveys of hospital staff during the 2020/21 season, it was found that only 39% of adults with vaccine-relevant underlying diseases, 23% of pregnant women and around 58% of medical staff were vaccinated in Germany [12], [13].

### Vaccination options for individuals <60 years of age

The available conventional influenza vaccines in Germany are quadrivalent inactivated, non-adjuvanted egg-based vaccines (QIVe) in standard doses, which are approved for individuals aged ≥6 months of age [14]. A nasal, egg-based live-attenuated vaccine (LAIV) is also available specifically for children and adolescents (2–17 years). The STIKO recommends LAIV as an equivalent option to conventional vaccines for this age group and specifically for individuals for whom an injection of conventional vaccines could lead to complications (e.g., coagulation disorders or injection phobia) [9]. Since November 2017, the STIKO has exclusively recommended quadrivalent vaccines that contain antigens for each of the following influenza strains: A(H1N1), A(H3N2), B(Victoria), and B(Yamagata) [15]. 

In Germany, a quadrivalent cell-based vaccine (QIVc) is also approved for individuals aged ≥2 years [14]. To address the persistent challenge of low effectiveness of conventional vaccines, influenza vaccines have been enhanced [15]. This enhancement is achieved through “stronger activation of humoral and cellular immunity” and/or “more constant antigenicity”, among other things. 

### Vaccine effectiveness (VE) of seasonal influenza vaccines

RKI estimates of VE against laboratory-confirmed influenza in Germany during the seasons 2012/13–2019/20 showed considerable fluctuations between the seasons, particularly for the A(H3N2) strain [16]. Furthermore, a global meta-analysis encompassing seasons 2004 to 2015 and consisting of 56 studies revealed that VE against A(H3N2) was considerably lower compared to VE against A(H1N1) and the B lineages. Specifically, in pediatric cohorts, VE against A(H3N2) was 43%, while in adult cohorts (≤60 and >60 years), it was 35% and 24%, respectively. In contrast, VE against A(H1N1) and the B lineages ranged between 62–73% and 54–63% across cohorts, respectively [17]. 

Interim data from six European studies of the 2022/23 season showed a variable VE against different influenza strains. The VE of available influenza vaccines ranged from 2–44% against A(H3N2), 28–46% against A(H1N1), and 64–85% against influenza B [18]. 

It is important to note that the VE of seasonal influenza vaccines depends on multiple factors, including the level of the match between vaccine and circulating viruses, the age and immunocompetence of the individual receiving the vaccine, their history of previous infections or vaccinations, the type of vaccine administered and the pathogenicity of the (dominant) circulating virus [15]. 

### Disadvantages of egg-based influenza vaccines

Egg-based vaccine production has disadvantages, including limited production capacity, prolonged production time and the rare occurrence of allergic reactions to egg-derived proteins [19]. Furthermore, influenza viruses have a high mutation rate, resulting in the selection of various variants best adapted to the avian host system when propagated in hen’s egg (a process known as egg adaptation). This egg adaptation leads to genetic differences between seed viruses used and vaccine viruses produced in eggs due to mutations at the receptor binding site of hemagglutinin (HA). Consequently, antigenic mismatch can occur between the vaccine viruses and circulating viruses, potentially impairing the effectiveness of egg-based influenza vaccines [19], [20]. Egg adaptation is observed in both influenza A and B viruses but is most often seen with A(H3N2) viruses [21]. Particularly egg adaptation in vaccine viruses of strain A(H3N2) show negative effects on VE [20].

### Cell-based influenza vaccines

To prevent egg adaptation of the viruses, cell-based vaccines are produced in mammalian cell cultures (Madin Darby Canine Kidney [MDCK] cells) [15], [21]. While antigen adaptions to the host system can still occur with cell-based production, mutations in the HA antigen are observed less frequently compared to propagation in chicken eggs [21]. This can result in improved VE compared to egg-based vaccines.

Since being approved, trivalent cell-based vaccines have been used in Germany for over 10 years. The quadrivalent formulation was approved in December 2018 [14] and has been administered in Germany starting from the 2019/20 season. In Europe, the use of QIVc is approved for individuals aged 2 years and older (Table 1 (Tab. 1)). Currently, there is an ongoing multicenter, randomized, controlled phase 3 trial (NCT03932682) examining the efficacy of QIVc versus non-influenza vaccine in infants (6–47 months) across multiple seasons. Immunogenicity and safety data from the 2019/20 season comparing QIVc with QIVe demonstrated that the QIVc was well tolerated in infants and not inferior to QIVe [22]. A study conducted using a 3-season (2017–2020) US exposure registry found no safety concerns associated with the use of QIVc in pregnant women aged 18-46 years, including those in their first trimester (n=665) [23].

## Results

### Effectiveness of the seasonal cell-based vaccine vs. conventional egg-based vaccines

Observational studies provide valuable insights, since VE is measured under real conditions (real-world data [RWD]). RWD are particularly relevant for influenza vaccines, as they allow measurement of VE fluctuations across multiple seasons and in various subgroups affected by virus and host factors (e.g., antigen drift, egg adaptation, age [immune senescence], comorbidities, vaccination status) [2]. RWD also enable the detection of rare side effects. Therefore, the inclusion of RWD serves as a valuable supplement to data from pivotal randomized controlled trials.

Below, we present RWD from retrospective cohort and case-control studies on the VE of QIVc versus QIVe during the US seasons 2017/18–2019/20. The dominant strains in each season were as follows: A(H3N2) in the 2017/18 season, co-circulation of A(H3N2) and A(H1N1) in the 2018/19 season, and A(H1N1) along with B(Victoria) in the 2019/20 season [24].

### Effectiveness of QIVc versus QIVe in preventing influenza-related outcomes in individuals ≥4 years of age over multiple consecutive seasons

A cohort study during the 2017/18 season, which involved individuals aged ≥4 years (n=1,353,862), demonstrated an overall relative vaccine effectiveness (rVE) of 36.2% (95% confidence interval [CI]: 26.1–44.9) for QIVc compared to QIVe in preventing influenza-like illness (ILI) [25]. Two cohort studies conducted during the 2018/19 (n=10,126,333) and 2019/20 (n=5,625,478) seasons compared the rVE of QIVc versus QIVe in the prevention of outpatient and inpatient medical encounters [26], [27]. The results showed a rVE of 7.6% (95% CI: 6.5–8.6) in individuals aged ≥4 years and 9.5% (95% CI: 7.9–11.1) in individuals aged ≥18 years, respectively. Notably, all studies showed a statistically significantly higher rVE in individuals under the age of 65. Furthermore, the rVE of QIVc compared to QIVe for the prevention of influenza-related hospitalizations/emergency department visits in individuals aged 4–64 years was analyzed in three cohorts during the 2017/18 (n=3,080,510), 2018/19 (n=3,727,890), and 2019/20 (n=5,065,326) seasons [28], [29], [30]. All three studies showed a statistically significantly higher rVE of QIVc compared to QIVe (2017/18: 14.4% [95% CI: 8.8–19.6], 2018/19: 6.5% [95% CI: 0.1–12.5], 2019/20: 5.3% [95% CI: 0.5–9.9]).

The rVE of QIVc compared to QIVe in preventing test-confirmed influenza was assessed in a case-control study that spanned the 2017/18–2019/20 seasons among individuals aged 4–64 years (n=99,610) [31]. Across all three seasons, QIVc demonstrated statistically significantly higher effectiveness with rVEs of 14.8% (95% CI: 7.0–22.0) in the 2017/18 season, 12.5% (95% CI: 4.7–19.6) in the 2018/19 season, and 10.0% (95% CI: 2.7–16.7) in the 2019/20 season.

### Influenza vaccination recommendations and options in selected countries

Some countries, such as Austria and the USA, recommend influenza vaccination for all individuals aged ≥6 months [32], [33]. In contrast, countries such as Germany, England, Italy, and Spain have specific recommendations for certain groups of people aged ≥6 months [9], [34], [35], [36]. Table 1 (Tab. 1) provides an overview of the current availability and preferred recommendations for influenza vaccines in selected countries.

QIVc is currently approved and available in all analyzed countries for individuals aged ≥2 years or ≥6 months, including the USA and the United Kingdom (since October 2023). In England, QIVc has received a preferential recommendation for individuals aged 18–64 years with a defined indication. In Germany, QIVc is recommended for individuals with an indication for vaccination that is equivalent to the egg-based vaccines available for the corresponding age groups. 

## Discussion

### Vaccination recommendations

In Germany, influenza is a prevalent respiratory disease that poses a substantial morbidity and mortality burden on the population. Given the significant direct and indirect costs associated with high morbidity rates among individuals below 60 years of age with influenza, it would be reasonable to consider expanding the STIKO vaccination recommendation to include healthy adults, children, and adolescents. This approach has already been implemented by the SIKO, the highest health authorities at the German state level, and in other countries, such as Austria and the USA. Currently, there are ongoing discussions within the STIKO regarding standard vaccination for children and preferential vaccine recommendations for specific indication groups [37]. 

### Egg adaptation

A significant drawback of egg-based influenza vaccine production is the occurrence of egg adaptation, which can result in reduced VE, especially against influenza A(H3N2). According to a consensus report, egg adaptation can lower vaccine matching by 7–21% and decrease VE by 4–16% [20]. Currently, the cell-based and recombinant vaccines are the only licensed influenza vaccines that are not produced in chicken eggs. However, it should be noted that the recombinant vaccine is currently unavailable in Germany (Table 1 (Tab. 1)). 

### Effectiveness and health economic data on the seasonal cell-based vaccine

Retrospective cohort studies during the US seasons 2017/18–2019/20, which included around 29 million participants, demonstrated a higher VE of QIVc compared to QIVe in preventing influenza-related outcomes among individuals aged 4–64 years [25], [26], [27], [28], [29], [30]. A limitation of these cohort studies is that the influenza cases were not laboratory-confirmed. However, the studies utilized influenza-specific ICD codes which have a high predictive value for laboratory-confirmed influenza. Some studies also conducted sensitivity analyses by focusing on the weeks with the highest influenza circulation and laboratory-confirmed cases. Additionally, according to the Infectious Diseases Society of America clinical practice guidelines, all hospitalized patients with influenza-like symptoms should be tested for influenza, indicating a high reliability of diagnostic codes [29], [30]. Moreover, a case-controlled study of approximately 100,000 individuals aged 4–64 years during the same influenza seasons in the US showed that QIVc was also more effective than QIVe in preventing test-confirmed influenza [31].

Lastly, a health economic evaluation demonstrated that using QIVc instead of QIVe in Germany could result in a reduction of influenza cases and complications and thereby generate cost savings from a societal perspective, making it a potentially cost-effective option [38].

## Conclusion

RWD of the US seasons 2017/18–2019/20 showed that QIVc demonstrated greater effectiveness compared to QIVe among individuals under the age of 65. The preferential use of QIVc could lead to a reduction in the influenza-related burden of disease and associated direct and indirect costs among the population under the age of 60 in Germany. 

### Key messages

- The population under the age of 60 contributes significantly to the disease burden and economic impact of seasonal influenza in Germany. Children are particularly vulnerable to severe cases of influenza and serve as carriers, transmitting the virus to adults and the elderly. Additionally, illness or caring for sick children leads to high productivity losses among the working population.
- Low influenza vaccination rates in Germany, combined with fluctuating effectiveness of available vaccines between seasons, result in suboptimal protection of the population against infections and severe illness.
- Conventional egg-based influenza vaccines can be affected by antigen adaptation, leading to mismatches between vaccine viruses and circulating strains, thus reducing vaccine effectiveness.
- Cell-based influenza vaccines are less susceptible to antigen adaptation and have shown significantly better effectiveness than conventional egg-based vaccines in the prevention of (test-confirmed) influenza infections and influenza-associated medical encounters under real-world conditions.
- Expanding vaccination recommendations to include all age groups and prioritizing cell-based over conventional egg-based influenza vaccines could reduce the burden of influenza-related illness and associated costs among the German population under the age of 60.

## Notes

### Acknowledgments

Mareike Konstanski from Xcenda GmbH provided background research and editorial support in preparing this manuscript, which was funded by CSL Seqirus GmbH.

### Annotation

The review has been published in German with the following bibliography:

Gärtner BC, Beier D, Gosch G, Wahle K, Wendt L, Förster LC, Schmidt KJ, Schwarz TF. Zellkulturbasierte Influenzaimpfstoffe: eine effektive Impfstoffoption für unter 60-Jährige. Wien Klin Wochenschr. 2024 Feb;136(Suppl 2):35-42. DOI: 10.1007/s00508-024-02327-3

### Authors’ ORCID

- Barbara C. Gärtner: 0000-0002-5234-7634
- Klaus Wahle: 0000-0002-2811-7150
- Laura-Christin Förster: 0009-0003-8557-9591
- Kim J. Schmidt: 0000-0001-8069-9103

### Competing interests

- CSL Seqirus GmbH funded the publication.
- Dr. Dietmar Beier: Advisory Board for CSL Seqirus.
- Prof. Dr. Barbara C. Gärtner: Advisory Board and honoraria for lectures for CSL Seqirus, Sanofi, GSK, Moderna, BioNTech, Pfizer, Viatris.
- Dr. Gunther Gosch: Lecturing activities and participation in advisory boards for AstraZeneca, CLS Seqirus, GSK, MSD, Pfizer, Sanofi Aventis.
- Prof. Dr. Klaus Wahle: Lectures and participation in advisory boards on behalf of or for SPMSD, CSL Seqirus, Pfizer, GSK, BioNTech.
- Dr. Luise Wendt: -
- Dr. Laura-Christin Förster is employed by CSL Seqirus GmbH.
- Kim J. Schmidt is employed by Xcenda GmbH, which received funding from CSL Seqirus GmbH for the preparation of this manuscript.
- Prof. Dr. Tino F. Schwarz: Honoraria for consulting and lectures for Alexion, AstraZeneca, Bavarian Nordic, Biogen, BioNTech, GSK, Janssen-Cilag, Merck-Serono, Moderna, MSD, Novavax, Pfizer, Roche, Sanofi-Aventis, CSL Seqirus, Synlab, Takeda and va-Q-tec.

## Figures and Tables

**Table 1:**
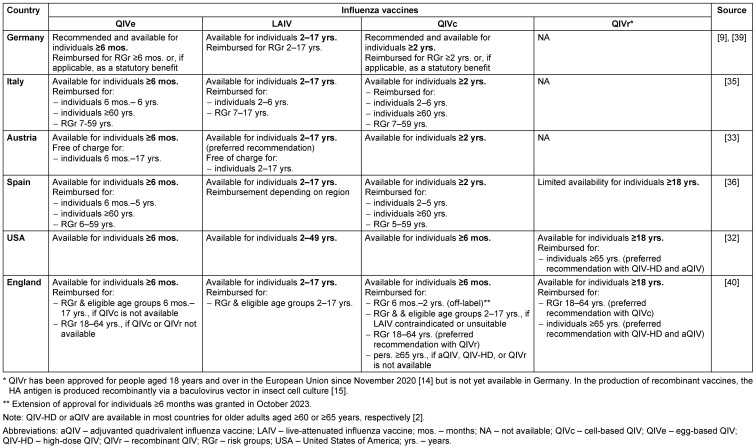
Availability and preferred recommendations of influenza vaccines in selected countries for persons ≥6 months in 2023/24 (modified based on [2])
